# Effects of radiotherapy on the survival of patients with stage IA and low-grade stage IB uterine endometrioid carcinoma

**DOI:** 10.1038/s41598-023-46435-y

**Published:** 2023-11-14

**Authors:** Shuqing Li, Zhihui Yi, Mingqing Li, Zhiling Zhu

**Affiliations:** Department of Obstetrics and Gynecology, Obstetrics and Gynecology Hospital of Fudan University, 128 Shenyang Road, Shanghai, 200090 People’s Republic of China

**Keywords:** Cancer, Medical research, Oncology

## Abstract

The present study aimed to evaluate the effects of radiotherapy on the overall survival of patients with primary stage IA, grade I–III uterine endometrioid carcinoma or stage IB, grade I–II uterine endometrioid carcinoma. A total of 7504 patients with stage IA, grade I–III uterine endometrioid carcinoma, and 857 patients with stage IB, grade I–II uterine endometrioid carcinoma were collected for the present study. Following propensity score matching (PSM), statistical analysis was performed for the equalized number of patients with stage IA, grade I–III uterine endometrioid carcinoma (n = 383) and patients with stage IB, grade I–II uterine endometrioid carcinoma (n = 330). For patients with primary stage IA, grade I–III uterine endometrioid carcinoma, radiotherapy was found to promoted a reduced 5-year overall survival rates [hazard ratio (HR), 1.726; 95% confidence interval (CI), 1.456–2.046; P < 0.05]. In patients with primary stage IB, grade I–II uterine endometrioid carcinoma, no significant differences were observed in the 5-year overall survival rates between radiotherapy and no radiotherapy groups (P = 0.059). In conclusion, radiotherapy may not improve 5-year overall survival for patients with primary stage IA, grade I–III or stage IB, grade I–II uterine endometrioid carcinoma.

## Introduction

Endometrial carcinoma is the most common gynecological malignancy in developed countries, and this may be a result of patients living longer and increasing rates of obesity^1,2^. Endometrial carcinoma is broadly classified into two major types, and these exhibit different clinicopathologic characteristics. Type 1 cancers are estrogen-driven, and comprise the majority of endometrial carcinomas. By contrast, type 2 cancers are less common, more aggressive and typically not estrogen-sensitive^3–5^. The prognosis of patients with endometrial carcinoma is determined primarily by histology, stage and grade. The majority of patients with endometrial carcinoma exhibit a favorable prognosis, due to endometrioid histology and early-stage disease^6,7^. Surgery is the main treatment option for endometrial carcinoma, including hysterectomy, salpingo-oophorectomy and lymphadenectomy^8^.

There is no consensus regarding the use of radiotherapy in patients with endometrial carcinoma that is considered to be confined to the uterus. Thus, distinguishing patients who may be successfully treated via surgery alone and those who exhibit significant risk of recurrence is challenging for gynecologists, and additional adjuvant therapy may be required. For the treatment of advanced-stage endometrial carcinoma, additional adjuvant therapy is often required; however, this is difficult to determine for patients with early-stage endometrial carcinoma^9,10^. At present, radiotherapy may increase psychological distress and the financial burden for patients and their families. Compared with patients who underwent surgery alone, those treated with radiotherapy prepared for a worse disease state. Side effects caused by radiotherapy, such as nausea, diarrhea, fecal leakage and lymphedema, severely impact the quality of life of patients. The majority of patients are required to monitor their diet, and some patients experience symptoms of depression and anxiety in association with these side effects^11,12^. Thus, the aforementioned side effects may exert a serious impact on the survival of patients, and numerous factors must be considered prior to radiotherapy.

Endometrioid carcinoma is the most common histologic type, which is diagnosed based on the evaluation of surgical specimens. The majority of endometrioid carcinomas are grades I and II, diagnosed at an early stage, and patients have a good prognosis^13,14^. At present, there is limited high-quality evidence specifically for endometrioid carcinoma highlighting that radiotherapy is associated with improved outcomes. Thus, the present study aimed to evaluate the effects of radiotherapy on the overall survival of patients with primary stage IA, grade I–III uterine endometrioid carcinoma or stage IB, grade I–II uterine endometrioid carcinoma.

## Methods

### Data sourcing

Data was extracted from the Surveillance, Epidemiology and End Results (SEER) database between 2010 and 2015. This database is the only comprehensive source of population-based information on cancer stage, therapy and survival in the United States.

### Inclusion criteria

Patients with primary stage IA, grade I–III uterine endometrioid carcinoma or stage IB, grade I–II uterine endometrioid carcinoma were identified. Patients were included in the present study following surgery, when chemotherapy had not been previously conducted. Information regarding tumor size and the number of lymph nodes removed was required. Notably, patients included in the present study were not positive for pelvic lymph nodes or para-aortic lymph nodes. Diagnosis depended on cytology or histology.

**Figure 1:**
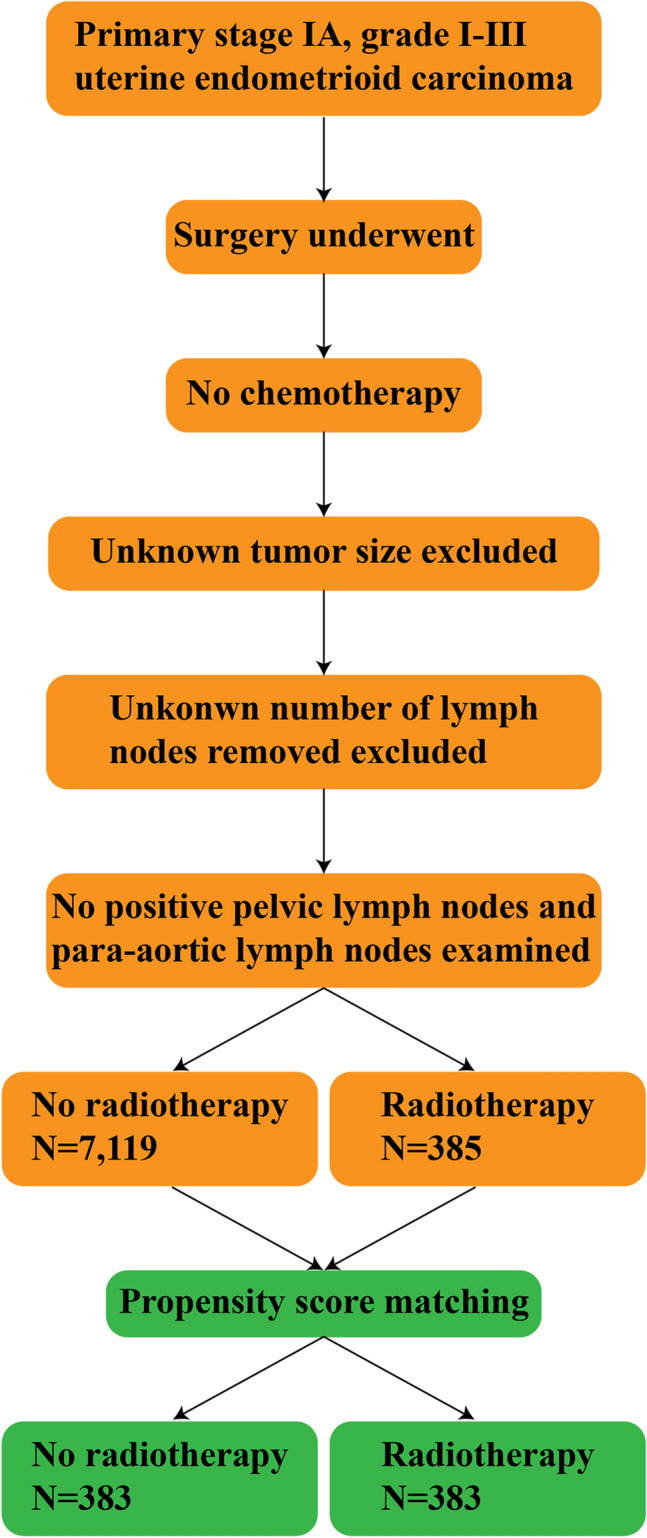
Flow chart for patients with primary stage IA, grade I–III uterine endometrioid carcinoma.

**Figure 2:**
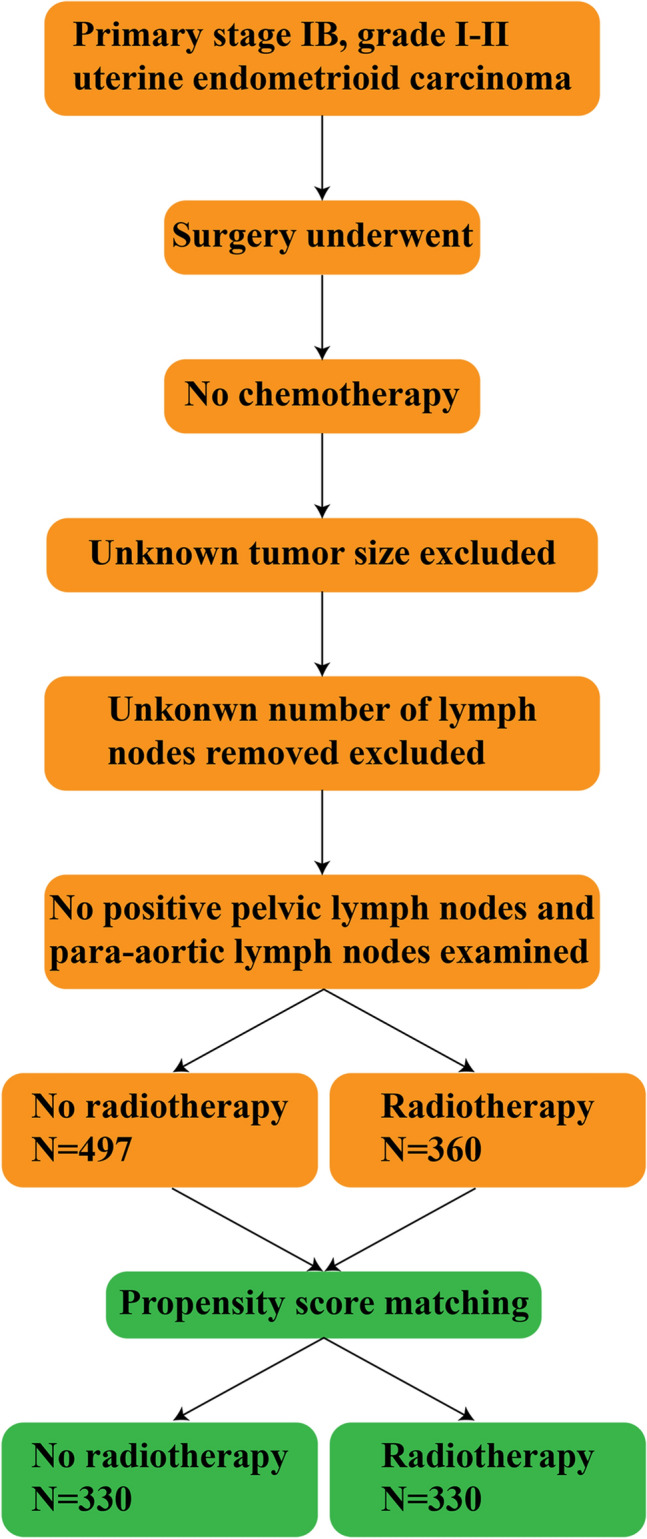
Flow chart for patients with primary stage IB, grade I–II uterine endometrioid carcinoma.

A total of 7504 patients with stage IA, grade I–III uterine endometrioid carcinoma and 857 patients with stage IB, grade I–II uterine endometrioid carcinoma were selected for inclusion in the present study. The following variables were measured: Radiotherapy, age, race, tumor grade, tumor size, regional lymph nodes removed and survival. In the extracted dataset, radiotherapy was categorized into two groups: (i) Yes and (ii) no; age was divided into two groups: (i) < 60 and (ii) ≥ 60 years; race was classified into two groups: (i) white and (ii) non-white; grade was categorized into three groups: (i) well differentiated, grade I; (ii) moderately differentiated, grade II; and (iii) poorly differentiated, grade III. In addition, tumor size was categorized into two groups: (i) < 2 and (ii) ≥ 2 cm; and regional lymph nodes were classified into two groups: (i) none and (ii) removed. All subjects were assessed for the association between radiotherapy and 5-year overall survival (Figs. 1 and 2).

### Statistical analysis

SPSS software (version 26; IBM Corp.) was used to perform statistical analysis. Univariate associations between categorical variables and radiotherapy were assessed using Pearson’s χ^2^-tests. All statistical tests were two-sided. P < 0.05 was considered to indicate a statistically significant difference. Propensity score matching (PSM) was used to minimize selection bias, which is common and inevitable in retrospective studies. Categorical variables included age, race, grade, tumor size and regional lymph nodes removed in both radiotherapy and no radiotherapy groups, and these were matched using a 1:1 match ratio method. Survival analysis was performed using the Kaplan–Meier method and log-rank tests. The Cox proportional hazards models were used to estimate the hazard ratio (HR) and 95% confidence interval (CI).

## Results

### Demographic features

There were 7504 patients (7119 who had not received radiotherapy and 385 who had) with primary stage IA, grade I–III uterine endometrioid carcinoma who met the study inclusion criteria. Among these patients, 49.5% were aged < 60 years and 50.5% were aged ≥ 60 years. A total of 84.2% of these patients were white. In addition, 74.4% of the patients were grade I, 22.9% were grade II and only 2.7% were grade III. A total of 38.8% of the patients had tumors measuring < 2 cm in size, whereas 61.2% had tumors measuring ≥ 2 cm. Regional lymph nodes were removed from only 4.7% of the patients, and 5-year overall survival rates were 72.6% (Table 1).

**Table 1:** Demographics for patients with stage IA uterine endometrioid carcinoma.

Characteristics	No	%
Radiotherapy		
No	7119	94.9
Yes	385	5.1
Age		
< 60 y	3716	49.5
≥ 60 y	3788	50.5
Race		
White	6319	84.2
Nonwhite	1185	15.8
Grade		
I	5584	74.4
II	1721	22.9
III	199	2.7
Tumor size		
< 2 cm	2910	38.8
≥ 2 cm	4594	61.2
Lymph node		
None	7155	95.3
Removed	349	4.7
Survival months		
< 60 m	2055	27.4
≥ 60 m	5449	72.6

There were 857 patients (497 who had not received radiotherapy and 360 who had) with primary stage IB, grade I–II uterine endometrioid carcinoma who met the study inclusion criteria. The majority of patients were aged ≥ 60 years (n = 660; 77.0%), and 87.7% of the patients were white. A total of 55.8% of the patients were grade I and 44.2% were grade II. Only 10.6% of the patients had tumors measuring < 2 cm in size, whereas 89.4% had tumors measuring ≥ 2 cm. Regional lymph nodes were removed from only 11.6% of the patients, and 5-year overall survival rates were 63.9% (Table 2).

**Table 2:** Demographics for patients with stage IB uterine endometrioid carcinoma.

Characteristics	No	%
Radiotherapy		
No	497	58.0
Yes	360	42.0
Age		
< 60 y	197	23.0
≥ 60 y	660	77.0
Race		
White	752	87.7
Nonwhite	105	12.3
Grade		
I	478	55.8
II	379	44.2
Tumor size		
< 2 cm	91	10.6
≥ 2 cm	766	89.4
Lymph node		
None	758	88.4
Removed	99	11.6
Survival months		
< 60 m	309	36.1
≥ 60 m	548	63.9

Data are expressed as n (%).

Laterality: the side of ovaries on which the primary tumor originated.

### Comparison of univariate categorical variables

The majority of patients with primary stage IA, grade I–III uterine endometrioid carcinoma in the radiotherapy groups were older than those in the no radiotherapy groups (< 60 years, 38.2 vs. 50.1%, respectively; and ≥ 60 years, 61.8 vs. 49.9%, respectively; P < 0.05). A higher number of patients were white in the radiotherapy groups (P = 0.405). A higher number of patients were grade II and grade III in the radiotherapy groups (grade I, 33.5 vs. 76.6%, respectively; grade II, 48.8 vs. 21.5%, respectively; and grade III, 17.7 vs. 1.8%, respectively; P < 0.05). Fewer patients had tumors measuring < 2 cm and an increased number of patients had tumors measuring ≥ 2 cm in the radiotherapy groups (< 2 cm, 15.8 vs. 40.0%, respectively; and ≥ 2 cm, 84.2 vs. 60.0%, respectively; P < 0.05). In addition, there were more patients with regional lymph nodes removed in the radiotherapy groups compared with the no radiotherapy group (11.2 vs. 4.3%, respectively; P < 0.05). Notably, selection bias and non-uniformity between the radiotherapy and no radiotherapy groups were eliminated using PSM. Statistical analysis was further performed for the equalized number of patients (n = 383). The categorical variables were well-balanced and the potential covariates between the two groups were markedly decreased (Table 3).

**Table 3:** Comparison of univariate covariates for patients with stage IA uterine endometrioid carcinoma.

Characteristics	Before PSM			After PSM		
	Radiotherapy− (n = 7119)	Radiotherapy+ (n = 385)	*P*	Radiotherapy− (n = 383)	Radiotherapy+ (n = 383)	*P*
Age			< 0.01			0.605
< 60 y	3569 (50.1)	147 (38.2)		154 (40.2)	147 (38.4)	
≥ 60 y	3550 (49.9)	238 (61.8)		229 (59.8)	236 (61.6)	
Race			0.405			0.458
White	5989 (84.1)	330 (85.7)		335 (87.5)	328 (85.6)	
Nonwhite	1130 (15.9)	55 (14.3)		48 (12.5)	55 (14.4)	
Grade			< 0.01			0.785
I	5455 (76.6)	129 (33.5)		138 (36.0)	129 (33.7)	
II	1533 (21.5)	188 (48.8)		180 (47.0)	188 (49.1)	
III	131 (1.8)	68 (17.7)		65 (17.0)	66 (17.2)	
Tumor size			< 0.01			0.027
< 2 cm	2849 (40.0)	61 (15.8)		85 (22.2)	61 (15.9)	
≥ 2 cm	4270 (60.0)	324 (84.2)		298 (77.8)	322 (84.1)	
Lymph node			< 0.01			0.468
None	6813 (95.7)	342 (88.8)		348 (90.9)	342 (89.3)	
Removed	306 (4.3)	43 (11.2)		35 (9.1)	41 (10.7)	

Fewer elderly patients with primary stage IB, grade I–II uterine endometrioid carcinoma tended to receive radiotherapy (< 60 years, 25.8 vs. 20.9%, respectively; and ≥ 60 years, 74.2 vs. 79.1%, respectively; P < 0.05). A higher number of patients were white in the radiotherapy groups (P = 0.851). Fewer patients were grade I, and an increased number of patients were grade II in the radiotherapy groups, compared with those in the no radiotherapy groups (grade I, 46.7 vs. 62.4%, respectively; and grade II, 53.3 vs. 37.6%, respectively; P < 0.05). Fewer patients had tumors measuring < 2 cm in size and an increased number of patients had tumors measuring ≥ 2 cm in the radiotherapy groups (< 2 cm, 6.1 vs. 13.9%, respectively; and ≥ 2 cm, 93.9 vs. 86.1%, respectively; P < 0.05). Fewer patients had regional lymph nodes removed in the radiotherapy groups compared with the no radiotherapy groups (P = 0.899). All categorical variables were well-balanced without significant differences following PSM. An equal number of patients were further analyzed (n = 330; Table 4).

**Table 4:** Comparison of univariate covariates for patients with stage IB uterine endometrioid carcinoma.

Characteristics	Before PSM			After PSM		
	Radiotherapy− (n = 497)	Radiotherapy+ (n = 360)	*P*	Radiotherapy− (n = 330)	Radiotherapy+ (n = 330)	*P*
Age			0.092			0.926
< 60 y	104 (20.9)	93 (25.8)		75 (22.7)	74 (22.4)	
≥ 60 y	393 (79.1)	267 (74.2)		255 (77.3)	256 (77.6)	
Race			0.851			1.000
White	437 (87.9)	315 (87.5)		293 (88.8)	293 (88.8)	
Nonwhite	60 (12.1)	45 (12.5)		37 (11.2)	37 (11.2)	
Grade			< 0.01			1.000
I	310 (62.4)	168 (46.7)		168 (50.9)	168 (50.9)	
II	187 (37.6)	192 (53.3)		162 (49.1)	162 (49.1)	
Tumor size			< 0.01			0.877
< 2 cm	69 (13.9)	22 (6.1)		23 (7.0)	22 (6.7)	
≥ 2 cm	428 (86.1)	338 (93.9)		307 (93.0)	308 (93.3)	
Lymph node			0.899			0.544
None	439 (88.3)	319 (88.6)		294 (89.1)	289 (87.6)	
Removed	58 (11.7)	41 (11.4)		36 (10.9)	41 (12.4)	

*PSM* propensity score matching. P-value < 0.05 is regarded as statistically significant.

### Univariate analysis of categorical variables and overall survival

The association between univariate categorical variables and 5-year overall survival rates was analyzed using the Kaplan–Meier method and log-rank tests. For patients with primary stage IA, grade I–III uterine endometrioid carcinoma, radiotherapy decreased 5-year overall survival rates compared with the no radiotherapy groups (69.5 vs. 81.7%, respectively; P < 0.05). There were no significant differences in 5-year overall survival rates between the < 60 and ≥ 60 years age groups (78.1 vs. 74.0%, respectively; P = 0.122). Grade was a risk factor for 5-year overall survival rates (grade I, 82.0%; grade II, 75.0%; and grade III, 64.1%, respectively; P < 0.05). Tumors < 2 cm in size were associated with higher 5-year overall survival rates compared with the tumor ≥ 2 cm groups (81.5 vs. 74.2%, respectively; P < 0.05). Notably, race and the removal of regional lymph nodes did not affect 5-year overall survival rates (P = 0.593 and P = 0.479; respectively; Table 5).

**Table 5:** Univariate analysis of clinical factors with 5-year overall survival for patients with stage IA uterine endometrioid carcinoma.

Characteristics	No	5-year OS (%)	*P*
Radiotherapy			< 0.01
No	313	81.7	
Yes	266	69.5	
Age			0.122
< 60 y	235	78.1	
≥ 60 y	344	74.0	
Race			0.593
White	499	75.3	
Nonwhite	80	77.7	
Grade			< 0.01
I	219	82.0	
II	276	75.0	
III	84	64.1	
Tumor size			< 0.01
< 2 cm	119	81.5	
≥ 2 cm	460	74.2	
Lymph node			0.479
None	524	75.9	
Removed	55	72.4	

For patients with primary stage IB, grade I–II uterine endometrioid carcinoma, 5-year overall survival rates were comparable between radiotherapy and no radiotherapy groups (64.5 vs. 65.2%, respectively; P < 0.05). Notably, age was a risk factor for 5-year overall survival rates, and these were 69.8 and 63.4% in < 60 and ≥ 60 years age groups, respectively (P < 0.05). There were no significant differences in 5-year overall survival rates between the grade I and grade II groups (71.1 vs. 58.3%, respectively; P = 0.446). In addition, tumors < 2 cm in size were associated with a higher 5-year overall survival rate, compared with the tumor ≥ 2 cm groups (77.8 vs. 63.9%, respectively; P < 0.05). Race and the removal of regional lymph nodes did not affect 5-year overall survival rates (P = 0.346 and P = 0.562; respectively; Table 6).

**Table 6:** Univariate analysis of clinical factors with 5-year overall survival for patients with stage IB uterine endometrioid carcinoma.

Characteristics	No	5-year OS (%)	*P*
Radiotherapy			0.033
No	215	65.2	
Yes	213	64.5	
Age			0.017
< 60 y	104	69.8	
≥ 60 y	324	63.4	
Race			0.346
White	374	63.8	
Nonwhite	54	73.0	
Grade			0.446
I	239	71.1	
II	189	58.3	
Tumor size			0.027
< 2 cm	35	77.8	
≥ 2 cm	393	63.9	
Lymph node			0.562
None	370	63.5	
Removed	58	75.3	

### Cox regression analysis

The correlation between the categorical variables and 5-year overall survival rates was further investigated using the univariate Cox regression analysis. For patients with primary stage IA, grade I–III uterine endometrioid carcinoma, radiotherapy decreased 5-year overall survival rates, compared with the no radiotherapy groups (HR, 1.883; 95% CI, 1.593–2.226; P < 0.05). A higher grade significantly reduced 5-year overall survival rates (grade II, HR, 1.763; 95% CI, 1.471–2.113; grade III, HR, 1.638; 95% CI, 1.272–2.110; P < 0.05). Tumors ≥ 2 cm in size were associated with a lower 5-year overall survival rate (HR, 1.704; 95% CI, 1.389–2.091; P < 0.05). Notably, age, race and the removal of regional lymph nodes exerted no influence on 5-year overall survival rates (P = 0.141, P = 0.608 and P = 0.503; respectively; Table 7).

**Table 7:** Univariate cox regression analysis for 5-year overall survival for patients with stage IA uterine endometrioid carcinoma.

Characteristics	HR (95% CI)	*P*
Radiotherapy		< 0.01
No	Ref	
Yes	1.883 (1.593–2.226)	
Age		0.141
< 60 y	Ref	
≥ 60 y	–	
Race		0.608
White	Ref	
Nonwhite	–	
Grade		< 0.01
I	Ref	
II	1.763 (1.471–2.113)	
III	1.638 (1.272–2.110)	
Tumor size		< 0.01
< 2 cm	Ref	
≥ 2 cm	1.704 (1.389–2.091)	
Lymph node		0.503
None	Ref	
Removed	–	

For patients with primary stage IB, grade I–II uterine endometrioid carcinoma, radiotherapy decreased 5-year overall survival rates compared with the no radiotherapy groups (HR, 1.220; 95% CI, 1.009–1.476; P < 0.05). Increased age reduced 5-year overall survival rates (HR, 1.296; 95% CI, 1.038–1.619; P < 0.05). In addition, tumors ≥ 2 cm in size were associated with a lower 5-year overall survival rate (HR, 1.450; 95% CI, 1.022–2.058; P < 0.05). Notably, race, grade and the removal of regional lymph nodes exerted no influence on 5-year overall survival rates (P = 0.357, P = 0.463 and P = 0.575; respectively; Table 8).

**Table 8:** Univariate cox regression analysis for 5-year overall survival for patients with stage IB uterine endometrioid carcinoma.

Characteristics	HR (95% CI)	*P*
Radiotherapy		0.040
No	Ref	
Yes	1.220 (1.009–1.476)	
Age		0.022
< 60 y	Ref	
≥ 60 y	1.296 (1.038–1.619)	
Race		0.357
White	Ref	
Nonwhite	–	
Grade		0.463
I	Ref	
II	–	
Tumor size		0.038
< 2 cm	Ref	
≥ 2 cm	1.450 (1.022–2.058)	
Lymph node		0.575
None	Ref	
Removed	–	

*HR* hazard ratios, *CI* confidence intervals, *Ref* reference. P-value < 0.05 is regarded as statistically significant.

To investigate how the categorical variables impacted 5-year overall survival in combination, these factors were incorporated into the multivariate Cox regression analysis. For patients with primary stage IA, grade I–III uterine endometrioid carcinoma, radiotherapy decreased 5-year overall survival rates (HR, 1.726; 95% CI, 1.456–2.046; P < 0.05). Notably, higher grades were associated with increased mortality rates (grade II, HR, 1.570; 95% CI, 1.304–1.890; grade III, HR, 1.315; 95% CI, 1.014–1.706; P < 0.05), and 5-year overall survival rates were reduced in the tumor ≥ 2 cm groups (HR, 1.372; 95% CI, 1.109–1.698; P < 0.05; Table 9).

**Table 9:** Multivariate cox regression analysis for 5-year overall survival for patients with stage IA uterine endometrioid carcinoma.

Characteristics	HR (95% CI)	*P*
Radiotherapy		< 0.01
No	Ref	
Yes	1.726 (1.456–2.046)	
Grade		0.039
I	Ref	
II	1.570 (1.304–1.890)	
III	1.315 (1.014–1.706)	
Tumor size		< 0.01
< 2 cm	Ref	
≥ 2 cm	1.372 (1.109–1.698)	

For patients with primary stage IB, grade I–II uterine endometrioid carcinoma, there were no significant differences in 5-year overall survival rates between the radiotherapy and no radiotherapy groups (P = 0.059). Increased age was associated with reduced 5-year overall survival rates (HR, 1.300; 95% CI, 1.040–1.623; P < 0.05). In addition, 5-year overall survival rates were reduced in the tumor ≥ 2 cm groups (HR, 1.455; 95% CI, 1.025–2.063; P < 0.05; Table 10).

**Table 10:** Multivariate cox regression analysis for 5-year overall survival for patients with stage IB uterine endometrioid carcinoma.

Characteristics	HR (95% CI)	*P*
Radiotherapy		0.059
No	Ref	
Yes	–	
Age		0.021
< 60 y	Ref	
≥ 60 y	1.300 (1.040–1.623)	
Tumor size		0.036
< 2 cm	Ref	
≥ 2 cm	1.455 (1.025–2.063)	

*HR* hazard ratios, *CI* confidence intervals, *Ref* reference. P-value < 0.05 is regarded as statistically significant.

## Discussion

Results of the present study demonstrated that 5-year overall survival was not improved following radiotherapy in patients with primary stage IA, grade I–III or stage IB, grade I–II uterine endometrioid carcinoma, who underwent surgery with no subsequent chemotherapy, and exhibited no positive pelvic lymph nodes or para-aortic lymph nodes. In accordance with results of the present study, Creutzberg et al. demonstrated that postoperative radiotherapy exerted no significant impact on overall survival in patients with stage IA and stage IB endometrial carcinoma^15^. Similarly, the results of previous randomized trials demonstrated that radiotherapy reduced the risk of local–regional recurrence, but did not improve overall survival for patients with early-stage endometrial carcinoma^16–18^.

The management of radiotherapy in early-stage endometrial carcinoma remains controversial. Rydzewski et al. reported that radiotherapy was associated with a reduction in overall mortality and an improvement in survival for patients with stage IA and IB endometrial carcinoma^19^. Similarly, results of a previous study demonstrated that radiotherapy significantly increased the survival of patients with stage IA and IB endometrial carcinoma^20^. Medenwald et al. revealed that radiotherapy in addition to surgery is beneficial for patients with stage IB endometrial carcinoma, but not for stage IA endometrial carcinoma^21^. These discrepancies may be due to study design, prediction of disease recurrence or prognosis. Moreover, these previous studies not only investigated endometrioid histology, but also other histological types. Although the majority of endometrial carcinomas include endometrioid histology, conclusions drawn in the present study cannot be applied to all patients with endometrioid histology. A stratified subgroup analysis of histological subtypes is required, and may demonstrate distinct differences in survival. In addition, previous studies including patients who received radiotherapy may lead to selection bias. Patients receiving radiotherapy may have improved health and financial status, which may impact survival time. Moreover, previous data was discounted in previous studies due to confounding factors, and the sample sizes analyzed were small; thus, statistical analyses may be impacted. Studies with improved designs and large-scale clinical trials are required to address the aforementioned limitations. In certain studies, the risk of disease recurrence could not be reliably predicted in early-stage endometrial carcinomas due to clinicopathologic features, such as histology, stage, grade or lymph node involvement; thus, these results may not be reproducible^22,23^. Therefore, the management of early-stage endometrial carcinomas remains controversial.

Results of the present study demonstrated that in patients with primary stage IA, grade I–III uterine endometrioid carcinoma, 5-year overall survival was markedly reduced by grade II/III and tumors ≥ 2 cm in size. Moreover, 5-year overall survival was significantly decreased following radiotherapy, not prospectively increased. For patients with primary stage IB, grade I–II uterine endometrioid carcinoma, age ≥ 60 years and tumors ≥ 2 cm in size were risk factors, which was consistent with the results of previous studies, and these factors reduced 5-year overall survival. Notably, no significant differences in 5-year overall survival were observed between the radiotherapy and no radiotherapy groups. Therefore, radiotherapy was not an appropriate treatment to improve 5-year overall survival for patients with primary stage IA, grade I–III or stage IB, grade I–II uterine endometrioid carcinoma, who underwent surgery and did not receive chemotherapy, and exhibited no positive pelvic lymph nodes or para-aortic lymph nodes. The aforementioned conclusion was drawn following the exclusion of all confounding factors. Notably, the risk of recurrence for early-stage uterine endometrioid carcinoma was low and prognosis was favorable. In addition, the side effects of radiotherapy may affect the survival of patients. Therefore, results of the present study may be encouraging.

The present study was based on a large-scale and comprehensive population-based cohort, and provided evidence on the efficacy of radiotherapy for treating primary stage IA, grade I–III and stage IB, grade I–II uterine endometrioid carcinoma. In addition, all subjects were randomly enrolled without subjective preference, which resulted in an objective conclusion.

However, the present study exhibits limitations. Notably, the SEER database lacked detailed information regarding lymph-vascular invasion. However, the risk of lymph-vascular invasion is low for early-stage endometrioid carcinoma^24^. Moreover, PSM was used to randomize the data and to decrease selection bias caused by this limitation.

In conclusion, radiotherapy may not improve 5-year overall survival for patients with primary stage IA, grade I–III or stage IB, grade I–II uterine endometrioid carcinoma. Further clinical trials are required to verify the conclusions of the present study.

## Data Availability

The datasets analyzed during the present study are available from the corresponding author on reasonable request.
